# Can transcranial direct current stimulation combined with interactive computerized cognitive training boost cognition and gait performance in older adults with mild cognitive impairment? a randomized controlled trial

**DOI:** 10.1186/s12984-024-01313-0

**Published:** 2024-02-16

**Authors:** Chi Ieong Lau, Mu-N Liu, Fang-Yu Cheng, Han-Cheng Wang, Vincent Walsh, Ying-Yi Liao

**Affiliations:** 1 Dementia Center, Department of Neurology, Shin Kong Wu Ho-Su Memorial Hospital, Taipei, Taiwan; 2 Applied Cognitive Neuroscience Group, Institute of Cognitive Neuroscience, University College London, London, UK; 3 Institute of Biophotonics, National Yang Ming Chiao Tung University, Taipei, Taiwan; 4 College of Medicine, Fu-Jen Catholic University, New Taipei City, Taiwan; 5 Department of Psychiatry, Taipei Veterans General Hospital, Taipei, Taiwan; 6 Division of Psychiatry, School of Medicine, National Yang Ming Chiao Tung University, Taipei, Taiwan; 7 Institute of Long-Term Care, MacKay Medical College, New Taipei, Taiwan; 8 College of Medicine, National Taiwan University, Taipei, Taiwan; 9 Department of Gerontological Health Care, National Taipei University of Nursing and Health Sciences, Taipei, Taiwan

## Abstract

**Background:**

Older adults with Mild Cognitive Impairment (MCI) are often subject to cognitive and gait deficits. Interactive Computerized Cognitive Training (ICCT) may improve cognitive function; however, the effect of such training on gait performance is limited. Transcranial Direct Current Stimulation (tDCS) improves cognition and gait performance. It remains unclear whether combining tDCS with ICCT produces an enhanced synergistic effect on cognition and complex gait performance relative to ICCT alone. This study aimed to compare the effects of tDCS combined with ICCT on cognition and gait performance in older adults with MCI.

**Method:**

Twenty-one older adults with MCI were randomly assigned to groups receiving either anodal tDCS and ICCT ( tDCS + ICCT ) or sham tDCS and ICCT ( sham + ICCT ). Participants played Nintendo Switch cognitive games for 40 min per session, simultaneously receiving either anodal or sham tDCS over the left dorsolateral prefrontal cortex for the first 20 min. Cognitive and gait assessments were performed before and after 15 training sessions.

**Results:**

The global cognition, executive function, and working-memory scores improved in both groups, but there were no significant interaction effects on cognitive outcomes. Additionally, the group × time interactions indicated that tDCS + ICCT significantly enhanced dual-task gait performance in terms of gait speed (*p* = 0.045), variability (*p* = 0.016), and dual-task cost (*p* = 0.039) compared to sham + ICCT.

**Conclusion:**

The combined effect of tDCS and ICCT on cognition was not superior to that of ICCT alone; however, it had a significant impact on dual-task gait performance. Administering tDCS as an adjunct to ICCT may thus provide additional benefits for older adults with MCI.

**Trial registration:**

This trial was registered at http://www.clinicaltrials.in.th/ (TCTR 20,220,328,009).

## Introduction

Mild Cognitive Impairment (MCI) is widely regarded as an intermediate stage between natural cognitive decline and the very early stages of dementia. One or more cognitive domains, such as executive function, working memory, and language can be impaired in patients with MCI, but their ability to perform the activities of daily living is often preserved [1]. In addition to cognitive deficits, older patients with MCI may experience gait dysfunction because gait control requires higher-level cognitive function, especially executive function. In elderly patients with MCI, limited cognitive capacity interferes with gait performance and increases the Dual-Task Cost (DTC) of cognitive–motor tasks [2]. Further, reduced gait speed and high gait variability lead to an increased risk of harmful falls in older patients with MCI [3]. However, to date there is no effective pharmacological treatment option available for MCI [4]. Due to the strong association between cognitive function and dual-task gait speed, finding treatment that improves both cognitive function and gait control ability in elderly patients with MCI is important [5].

Cognitive rehabilitation is a feasible nonpharmacological intervention for elderly patients with MCI [6]. Cognitive rehabilitation procedure commonly used for MCI include cognitive stimulation, memory-based interventions, multidomain approaches, and compensatory strategies [7]. Memory and multidomain interventions have demonstrated potential benefits, with memory-based approaches possibly being more effective [8]. Furthermore, some studies reported improved cognitive performance and reduced brain activation following training, while others did not observe these training-induced brain plasticity changes, despite improved cognitive performance after 5 weeks of working memory training in elderly adults with MCI [9, 10]. With advancements in computing technology, researchers have been able to conduct Computerized Cognitive Training (CCT). However, a systematic review has found that CCT and rehabilitation yield promising but inconsistent effects [11]. Recently, game companies have incorporated interactive technology into computerized exergames, such as those available on XBOX Kinect or Nintendo Switch. Evidence suggests that this form of Interactive Computerized Cognitive Training (ICCT) improves cognitive function in elderly patients with MCI [12, 13]. Given that cognitive function is a determinant of dual-task gait parameters [14], it is possible that improved cognitive function resulting from ICCT may also lead to enhanced dual-task gait performance. However, there is insufficient evidence to demonstrate the impact of ICCT on dual-task walking performance in elderly adults with MCI.

Transcranial Direct-Current Stimulation (tDCS) is a noninvasive technique that regulates brain excitability by delivering a low electrical current intensity (0.5–2.0 mA) through the scalp [15]. The administration of tDCS may cause changes in the membrane potential and modulate postsynaptic firing [16]. The Dorsolateral Prefrontal Cortex (DLPFC) is involved in high-level cognitive function. A growing body of research supports the effectiveness of applying anodal tDCS over the DLPFC to enhance cognitive performance, including processing speed, working memory, and executive function, in elderly with MCI [17, 18]. However, some studies suggest that tDCS did not demonstrate additional benefits on cognition in older adults [19]. A systematic review indicated that tDCS appears to have a mild positive effect on memory and language in the elderly with MCI [20]. Specifically, the DLPFC is the area responsible for allocating cognitive resources between two tasks performed simultaneously, and it plays an essential role in dual-task gait control [21]. Studies have shown that dual-task gait performance (e.g., stride time and variability) in elderly people can be improved immediately after the application of tDCS over the DLPFC [22, 23].

Despite this, the question of whether the combination of tDCS with cognitive training yields a better result than cognitive training alone remains unresolved. One study with an intervention period of 9–12 sessions found that this combination was not better than cognitive training alone on cognitive function [24]. In contrast, another study combining tDCS with memory training over 12 sessions showed significantly greater improvement in delayed recall compared to the tDCS alone or memory training groups [25]. The uncertainty regarding cognitive outcomes and whether the combination has a synergistic effect on dual-task performance compared to ICCT alone remains unresolved and requires further investigation. The novelty of this study lies in examining the synergistic effects of these two training approaches and expanding the scope of outcomes from cognition to dual-task gait performance. Therefore, our aim was to compare the effects of tDCS combined with ICCT (tDCS + ICCT) to those of sham tDCS with ICCT (sham + ICCT) on dual-task walking and cognitive function in elderly patients with MCI. Our hypothesis was that tDCS + ICCT would exert greater synergistic effects in these patients compared to sham + ICCT.

## Methods

### Participants

We recruited patients from the Dementia Center, Shin-Kong Wu Ho‐Su Memorial Hospital, Taiwan, to participate in this study. Out of 45 potential candidates, 22 provided their informed consent and were subsequently enrolled in the study. Patients were included in the study if they met the following criteria: (1) were aged at least 65 years; (2) had been diagnosed with MCI according to Petersen’s criteria [4] ; (3) had received a global rating of 0.5 on the Clinical Dementia Rating (CDR) scale; and (4) could walk at least 10 m unaided. Patients were excluded if they had: (1) dementia; (2) a cerebral tumor; (3) a history of cerebral infarction or hemorrhage; (4) any other known neurodegenerative or neuropsychiatric condition; (5) any orthopedic disease that would make participation in the study difficult; or (6) fewer than six years of education (elementary school).

### Study design

We conducted the study as a randomized controlled trial and ensured double-blinding (both raters and participants). Participants were randomly assigned to either the tDCS + ICCT group or the sham + ICCT group using numbers placed in sealed envelopes. Training was administered three times a week for five weeks, totaling 15 sessions. Each session lasted for 40 min of Switch training. The tDCS group received stimulation during the initial 20 min of each session, while the sham group underwent stimulation for 30 s at the start of the session. The participants were trained individually by an experienced research assistant. Each participant also underwent baseline assessments one day before the intervention and post assessments one day after the intervention, all conducted by a rater who was unaware of the group assignments. The rater performed two independent tests: a cognitive assessment followed by a gait assessment. The study protocol was approved by the ethics committee of Shin-Kong Wu Ho‐Su Memorial Hospital (IRB number: 20200709D, August 28, 2020). Additionally, this experiment is registered as a clinical trial at http://www.clinicaltrials.in.th/ (TCTR 20,220,328,009).

### tDCS

**Fig. 1:**
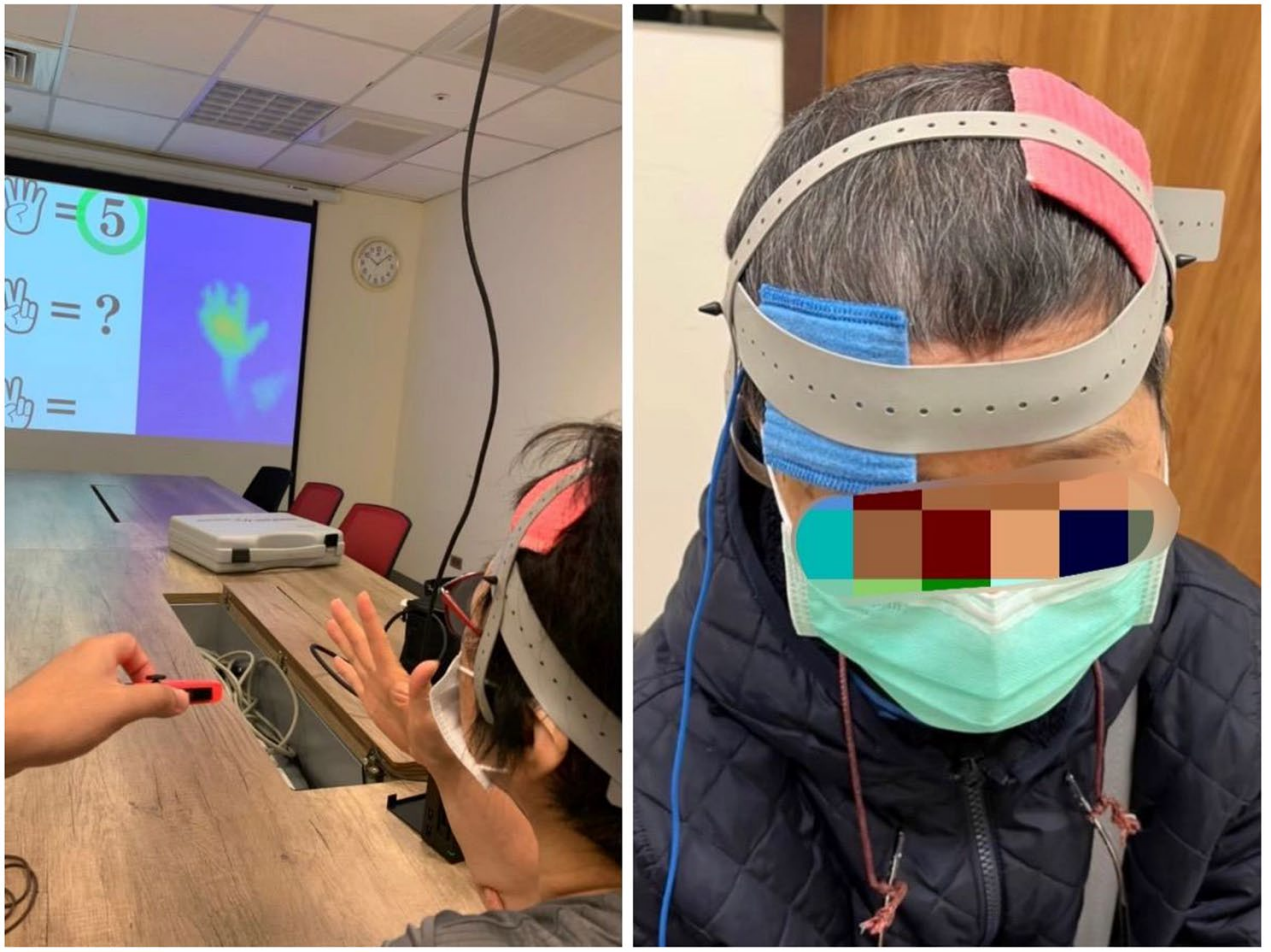
Left: example of a subject receiving the tDCS + ICCT protocol. Right: the tDCS montage

For the stimulation, we utilized a battery-operated constant DC Stimulator Plus (NeuroConn, Ilmenau, Germany). The stimulation delivered consisted of 2 mA of current via a pair of sponge electrodes soaked in a saline solution. These electrodes measured 50 × 70 mm (3,500 mm²) in size and were positioned to deliver optimal stimulation to the left DLPFC. Specifically, we placed the anode centrally over the F3 position according to the international 10–20 system, and the cathode was positioned at Fp4, corresponding to the contralateral right supraorbital region, as illustrated in Fig. 1. In the tDCS + ICCT group, this direct current was administered continuously for 20 min, following a 10-second ramp-up period. The duration of 20 min is considered both safe and commonly used in clinical research [26, 27]. The stimulation parameters and electrode montage for the sham + ICCT group were identical to those in the tDCS + ICCT group, with the exception that the current was only delivered for 30 s and then gradually reduced to 0 mA. This approach aimed to replicate the tingling sensation associated with active stimulation, making it indistinguishable from anodal tDCS treatment, although we assume it had negligible effects on the participants’ brains. We monitored the impedance of the electrodes throughout the entire stimulation period in all sessions to ensure that it remained below 5 kΩ for safety.

### ICCT program

The ICCT program covered executive function, attention, and memory domain and was administered via a commercial virtual-reality game console developed by Nintendo Switch in 2017. Nintendo Switch games can be played either on the console’s own screen or on a linked television screen. The participants used Switch Joy-Cons, which have infrared motion sensors on both sides, to detect their hand movements. We selected the following interactive games from the game library to use for cognitive training. During each session, all seven games we selected were played. Participants consistently began at the foundational level, with the program automatically adjusting difficulty based on their performance throughout the 40-minute duration.

1. Finger Calculation: A picture on the screen showed a number of fingers held up on two hands. The participants had to calculate the sum and mimic the number with their own fingers in front of the Joy-Con camera as quickly as possible.
2. Finger Gymnastics: The screen displayed a series of rock-paper-scissors graphics. The participants had to imitate the displayed graphics in front of the camera as quickly as possible.
3. Rock, Paper, Scissors Test: A picture of a hand in the rock, paper, or scissors position appeared randomly on the screen. The participants had to respond with the appropriate gesture to win the round according to the standard rock, paper, scissors rules.
4. Bird and Box Counting: The participants had to calculate the number of birds or boxes illustrated on the screen and select the correct number with the Joy-Con.
5. Working Memory: Groups of pictures appeared randomly on the screen. The participants had to select the picture that had appeared in the previous group from three pictures on the next screen. To increase the difficulty level, the participants had to select the photo that had appeared two rounds previously.
6. Five in a Row: On a grid on which pieces can be placed at the intersections of horizontal and vertical lines, the participant had to try to make a horizontal, vertical, or diagonal line of exactly five pieces before the computer did so.
7. Card Memory Test: Twenty playing cards were displayed face-down on the screen. The participant and the computer took turns turning over single cards and then replacing them face-down again. The participant had to try and memorize these. The one whose turn it was had to try and find another card that matched the one turned over (based on their memory of previously revealed cards). If they found one, they would take both cards. The game finished when all the face-down cards had been turned over and taken.

### Outcome measures

#### Cognitive performance

1. Global cognitive function

We assessed the participants’ global cognitive function using the Montreal Cognitive Assessment (MoCA). In the MoCA, the total score ranges from 0 to 30, with a higher score indicating better global cognitive function [28].

2. Working memory

We evaluated participants’ working memory using the N-back task. In this test, the participants had to indicate whether the current position matched the one used one step previously (1-back) and two steps previously (2-back) in the sequence. We tallied the number of trials the participant answered correctly [29]. The participants also completed the visual working memory (VWM) test. In this test, three colored squares were randomly displayed for 100 ms on a screen. After a retention interval of 900 ms, another set of colored squares was displayed. The participant was asked to indicate whether the current display was identical in color to the previous one. We calculated the reaction time as d-prime (d’) [30].

3. Episodic memory

We assessed the participants’ episodic memory using the Chinese Version of the Verbal Learning Test (CVVLT). Nine nouns were recited and the participants had to attempt to recall them immediately and again after a delay of 15 min. The number they recalled correctly was recorded, and this process was repeated four times [31].

4. Executive function

We assessed the participants’ attention, task-switching ability, and executive function using the Trail Making Test (TMT) parts A and B. In TMT-A, the participant had to connect 25 numbers in the correct sequence, and in TMT-B, they had to link 12 numbers with the 12 signs of the zodiac in the correct order as quickly as possible [32]. We recorded the time the participant took to complete each test. We also assessed their executive function using the Tower of London (ToL) task. The participant had to move colored beads arranged on three vertical rods to achieve a particular goal arrangement. Successive tasks increased in complexity and the participant had to perform two to five moves to reach the goal in each case. We analyzed the total time they took and their accuracy [33].

#### Gait performance

We measured the participants’ gait parameters using the Gait Up system (Gait Up, Lausanne, Switzerland). The system consists of a wearable device, and the measurement instrument has good validity and reliability [34]. GaitUp utilizes two wireless inertial sensors equipped with triaxial accelerometers, along with one tablet. The sampling frequency of the Gait Up system is 128 Hz. These sensors are secured using two rubber clips, attached to the dorsal side of the participants’ shoes, just below the lateral malleolus. The sensors can establish a wireless connection with a tablet for data control. In each trial, upon pressing “start” on the tablet, a three-second countdown is displayed before the “go” signal, during which the sensors undergo automatic calibration. The walking path was a straight corridor with 10 m marked as the starting and finishing points for each trial. The system automatically excludes the first and last gait cycles (acceleration and deceleration) and then computes the average gait parameters during the 10 m. A PDF report is instantly generated and uploaded to the cloud after participants complete each trial. We set up two tasks to assess the participant’s gait performance: (1) a single task, involving walking at the participant’s preferred speed; (2) a dual task, involving walking while conducting a subtraction task, in which the participant had to start from a randomized three-digit number and subtract three from it serially (e.g., 100, 97, 94 …). We asked the participant to perform three trials of each task and recorded the following spatiotemporal parameters: speed (m/s), stride length (m), and cadence (step/min). We took the average of each parameter from the measurements made during the three trials. We quantified dual-task interference based on the DTC of speed, calculated as: DTC of speed (%) = [single-task walking speed – dual-task walking speed) / single-task walking speed] x 100% [35]. Finally, we defined gait variability as the coefficient of variation of stride length (i.e., standard deviation/mean × 100%).

### Data analysis

We analyzed the sociodemographic, neuropsychological, and gait data using SPSS 20.0 (SPSS, Chicago, IL, USA). Descriptive statistics were generated for all variables, and distributions were expressed as means ± standard deviations or as counts. Initially, we assessed the uniformity of data distribution. Next, between-group differences in baseline characteristics were examined using independent t-tests or chi-squared tests. To evaluate the effects of ICCT on participants’ cognitive function, we conducted a two-way repeated-measures analysis of variance (ANOVA). The model included two groups, the timepoint of assessment (before or after the intervention), and the interaction between group and timepoint. Post-hoc analyses were performed with Bonferroni correction. Statistical significance was defined as *p* < 0.05. Partial eta-squared (η2) was calculated and used to indicate the effect size.

## Results

**Fig. 2:**
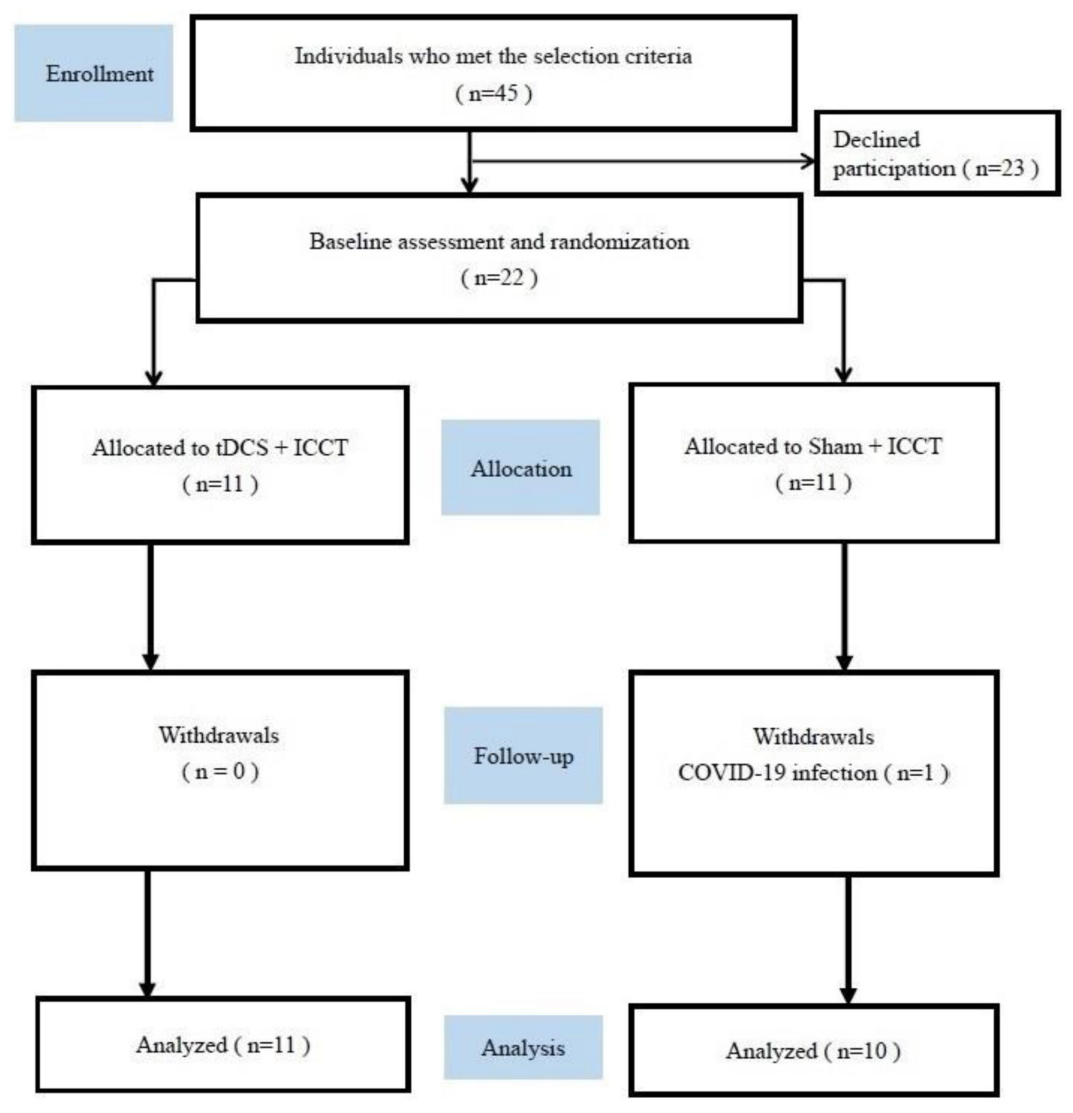
Flowchart of this study

The sample size we initially calculated for our study was 40 participants, based on an effect size of 0.23, an alpha level of 5%, and a power of 80% with a repeated measures ANOVA model [36]. However, our study coincided with the COVID-19 pandemic, which resulted in interruptions and patient reluctance to undergo interventions at the hospital. As a result, we were only able to enroll 22 patients who were then randomly assigned to one of the two groups (Fig. 2). Of these participants, one member of the sham + ICCT group dropped out of the study because of COVID-19 (she completed 9 of the 15 sessions). We, therefore, excluded this participant’s data from the analysis. The remaining 21 participants successfully completed all interventions and assessments, and none of them reported any adverse events. We conducted a brief post-intervention interview, which indicated that all the participants were confident that they had received the real tDCS treatment. The demographic characteristics of the patients are shown in Table 1. The results of the cognitive function tests and dual-task gait performance assessments, both before and after training, are presented in Tables 2 and 3, respectively. We observed no significant differences in demographic parameters between the two groups. Additionally, there were no significant differences in all cognitive and gait parameters between the two groups at baseline.

**Table 1:** Baseline demographic characteristics of the participants (*n* = 21)

	Sham + ICCT (*n* = 10)	tDCS + ICCT (*n* = 11)	*p*
Age (years)	69.0 ± 4.9	72.0 ± 17.3	0.606
Sex (female/male)	7/3	7/4	0.562
Education (years)	12.0 ± 4.9	11.8 ± 3.5	0.949
Height (cm)	157.9 ± 8.6	161.5 ± 6.5	0.286
Body weight (kg)	58.1 ± 7.9	58.7 ± 10.4	0.889
Body mass index (kg/m^2^)	23.2 ± 1.9	22.4 ± 3.2	0.476
MMSE (score)	26.0 ± 2.7	24.8 ± 1.4	0.228
CDR (score)	0.5 ± 0	0.5 ± 0	1

MMSE: Mini–Mental State Examination; CDR: Clinical Dementia Rating; Data are presented as means ± SD or numbers

**Table 2:** Comparisons of cognitive performance between the two treatment groups

	Sham + ICCT (*n* = 10)		tDCS + ICCT (*n* = 11)		Time	Group	Time × Group
	Pre- intervention	Post- intervention	Pre- intervention	Post- intervention	*P* value	*P* value	*P* value,η^2^
MoCA (score)	25.5 ± 2.7	26.6 ± 2.2*	25.0 ± 2.2	26.6 ± 1.7*	< 0.001	0.849	0.351, 0.046
TMTA (seconds)	63.3 ± 8.2	52.5 ± 10.3	68.3 ± 7.6	48.3 ± 8.9*	< 0.001	0.899	0.058, 0.177
TMTB (seconds)	111.6 ± 24.0	92.8 ± 19.4*	124.6 ± 39.5	91.4 ± 32.5*	< 0.001	0.645	0.128, 0.118
Tower of London							
Total time (seconds)	725.8 ± 175.2	693.2 ± 136.9	703.4 ± 242.3	587.4 ± 331.6	0.160	0.486	0.422, 0.034
Accuracy	6.5 ± 2.3	6.8 ± 1.6	6.1 ± 2.5	7.1 ± 2.4	0.094	0.973	0.354, 0.045
VWM (d’)	1.5 ± 0.3	1.5 ± 0.4	1.8 ± 0.9	1.7 ± 0.9	0.430	0.388	0.859, 0.002
CVVLT (number)							
Verbal memory	24.8 ± 4.7	28.0 ± 3.9*	23.3 ± 4.9	28.3 ± 5.6*	< 0.001	0.799	0.120, 0.122
Delayed recall	4.1 ± 2.0	5.3 ± 2.2	3.6 ± 2.4	5.2 ± 2.5*	0.003	0.797	0.599, 0.015
N-back task (number)							
1-back	15.2 ± 0.7	17.9 ± 1.5*	14.1 ± 0.9	18.3 ± 1.7*	< 0.001	0.532	0.067, 0.166
2-back	6.1 ± 2.2	7.1 ± 2.8	4.8 ± 1.8	7.3 ± 2.0*	< 0.001	0.581	0.070, 0.162

Data are presented as means ± SD or numbers. *significant pre-post improvement by the post-hoc comparison with Bonferroni correction

MoCA : Montreal Cognitive Assessment; TMTA and TMTB: Trail Making Test parts A and B; VWM: Visual Working Memory; CVVLT: Chinese Version of the Verbal Learning Test

**Table 3:** Comparisons of walking performance between the two treatment groups

	Sham + ICCT (*n* = 10)		tDCS + ICCT (*n* = 11)		Time	Group	Time × Group effect
	Pre- intervention	Post- intervention	Pre- intervention	Post- intervention	*P* value	*P* value	*P* value,η^2^
Single task walking							
Speed (m/s)	1.1 ± 0.2	1.0 ± 0.1	1.1 ± 0.2	1.1 ± 0.2	0.253	0.778	0.461, 0.029
Cadence (step/min)	111.5 ± 20.6	112.5 ± 19.8	111.0 ± 13.1	119.4 ± 15.3	0.291	0.616	0.400, 0.037
Stride length (m)	1.0 ± 0.1	1.0 ± 0.1	1.0 ± 0.2	1.1 ± 0.2	0.369	0.343	0.102, 0.134
Gait variability	3.5 ± 1.0	3.6 ± 1.1	3.7 ± 0.9	3.1 ± 0.9	0.334	0.757	0.164, 0.100
Dual task walking							
Speed (m/s)	0.8 ± 0.3	0.9 ± 0.3*	0.8 ± 0.4	1.0 ± 0.2*	0.009	0.811	0.045, 0.195
Cadence (step/min)	96.3 ± 24.5	102.2 ± 23.3	85.5 ± 32.1	111.9 ± 17.6*	0.004	0.953	0.050, 0.188
Stride length (m)	0.9 ± 0.1	0.9 ± 0.1	0.9 ± 0.2	1.0 ± 0.2	0.220	0.245	0.123, 0.121
Gait variability	4.0 ± 1.20	4.1 ± 1.51	4.5 ± 1.41	3.4 ± 1.08*	0.043	0.881	0.016, 0.271
DTC of speed (%)	19.6 ± 30.7	14.3 ± 25.4	30.6 ± 30.7	6.9 ± 15.7*	0.004	0.846	0.039, 0.206

Data are presented as means ± SD or numbers. * significant pre-post improvement by the post-hoc comparison with Bonferroni correction

DTC: Dual Task Cost

In Table 2, no significant interaction and group effect were observed for any of the cognitive function outcomes. However, there was a significant time effect on MOCA (*p* < 0.001), TMTA (*p* < 0.001), TMTB (*p* < 0.001), CVVLT verbal memory (*p* < 0.001), CVVLT delayed recall (*p* = 0.003), 1-back (*p* < 0.001), and 2-back (*p* < 0.001). In the tDCS + ICCT group, the post-hoc comparisons with Bonferroni correction showed that participants’ scores for the MoCA (*p* = 0.002), TMTA (*p* < 0.001), TMTB (*p* = 0.002), CVVLT verbal memory (*p* < 0.001) and delayed recall (*p* < 0.001), 1-back task test (*p* < 0.001), and 2-back task test (*p* < 0.001) improved significantly after training. In the sham + ICCT group, the post-hoc comparisons with Bonferroni correction showed that participants’ scores for the MoCA (*p* = 0.014), TMTB (*p* = 0.012), CVVLT verbal memory (*p* = 0.008), and 1-back task test (*p* = 0.002) improved significantly after training.

In Table 3, significant interaction effects were observed in relation to gait speed (*p* = 0.045, η^2^ = 0.195), variability (*p* = 0.016, η^2^ = 0.271), and DTC (*p* = 0.039, η^2^ = 0.206) during dual-task walking, with the tDCS + ICCT group performing better than the sham + ICCT group. There was no observed group effect, but time effects were detected in speed (*p* = 0.009), cadence (*p* = 0.004), gait variability (*p* = 0.043) during dual task walking, and DTC of speed (*p* = 0.004). In the tDCS + ICCT group, the post-hoc comparisons with Bonferroni correction showed that gait speed (*p* = 0.046), cadence (*p* = 0.020), variability (*p* < 0.001), and DTC during dual-task walking (*p* = 0.018) improved significantly after training. In the sham + ICCT group, the post-hoc comparisons with Bonferroni correction showed that gait speed (*p* = 0.046) during dual-task walking improved significantly after training.

## Discussion

In the current study, we compared the effects of administering anodal tDCS combined with ICCT to those of administering ICCT alone on cognitive function and dual-task gait performance. Both groups showed improved global cognitive function, executive function, and working memory after the training. However, the effects of tDCS combined with ICCT were not superior to ICCT alone in terms of cognitive function because no significant nteraction effects were found. Furthermore, a significant interaction effect was found in some dual-task parameters, indicating that the tDCS + ICCT treatment was superior to ICCT alone in improving dual-task walking performance.

A meta-analysis revealed that tDCS may improve memory and language in elderly patients with MCI [20]. However, our findings revealed no synergistic effect of the tDCS–ICCT combination on all cognitive outcomes compared to ICCT alone. This may be because both types of training enhanced cognitive function. Our brain-training Nintendo Switch program includes many interactive and fun composite cognitive training. Given the available evidence, which suggests that CCT has moderate effects on various aspects of cognitive function, and that tDCS has a mild positive influence on memory and language skills in older adults with MCI, it is reasonable to consider the possibility that the training effect of ICCT was superior to that of tDCS on enhancing cognitive performance [20, 37]. Our results thus agree with previous findings that combining cognitive training with either anodal or sham tDCS on the DLPFC may significantly improve cognitive function, but that adding anodal tDCS would lead to no significant between-group differences in older adults with MCI [24, 38].

However, we observed a significant pre-post improvement in working memory in the tDCS + ICCT group, particularly in the CVVLT delayed recall and 2-back tasks, which was not observed in the sham + ICCT group. Furthermore, time by group interaction effects were also marginally significant in the TMT-A and N-back tasks, suggesting the potential of the tDCS + ICCT treatment to be more beneficial than sham + ICCT for cognitive function. Recent studies have suggested that concurrent tDCS stimulation during cognitive training tasks could enhance neural plasticity in the cortical regions that are stimulated [39, 40]. Considering that our ICCT program includes memory training games similar to the N-back tasks, it’s possible that tDCS had synergistic effects on this specific cognitive domain. This is supported by the improvements observed in the two corresponding working memory tasks (the 2-back and CVVLT delayed recall tasks) in the tDCS + ICCT group, which were not seen in the sham + ICCT group. However, it’s important to mention that our study consisted of just 15 training sessions. Future research could explore whether increasing this number yields significantly different outcomes between the treatment groups.

In addition to investigating the combined effects of tDCS + ICCT on cognitive performance, we also examined their impact on gait, a parameter not previously explored in studies [24, 25, 38]. Walking and simultaneously performing serial subtraction require processing and filtering out unrelated signals. Therefore, this task is highly demanding for older adults with MCI [41, 42]. Traditionally, most studies have involved longer cognitive-motor dual-task training (24 sessions over 12–24 weeks) to improve dual-task gait performance [43, 44]. However, our findings show that training effects can be seen after as little as five weeks (15 sessions) when combining tDCS with ICCT. This implies that applying tDCS during cognitive training may be more effective than using cognitive–motor dual-task training for improving dual-task gait performance, especially with respect to speed, DTC, and variability. Further, ICCT alone may not be sufficient to improve dual-task gait performance, or a higher intensity and duration may be required to achieve the same amount of improvement. The immediate impact of tDCS in reducing DTC has been established in healthy elderly individuals, whether administered before or during complex walking. Our study extends this understanding to multiple sessions and incorporates elderly individuals with MCI [21, 45].

The left DLPFC is a brain region responsible for executive function, allocating cognitive resources, and potentially reducing dual-task walking costs in older adults [46, 47]. Patients with MCI exhibit attenuated prefrontal cortex activation during dual-task walking [48, 49]. Further, studies have shown greater activation of the left DLPFC during cognitive–motor walking tasks than during single-task walking [50, 51]. Our findings of increased dual-task gait speed and reduced variability following the additional administration of tDCS over the left DLPFC with ICCT provide further evidence to support the crucial role in gait control played by DLPFC.

We hypothesized tha ICCT may improve the overall cognitive capacity, and thus allow more attentional resources to be devoted towards a walking task while performing a cognitive task simultaneously. In our finding, the decrease in DTC in the tDCS + ICCT group may indicate that more attentional resources are available for walking during dual-task walking task, potentially due to an increased overall cognitive capacity. This implies that tDCS enhanced neural efficiency by modulating prefrontal recruitment. Support for this idea comes from a recent study that demonstrated a decrease in left prefrontal oxygen consumption, corresponding to a reduction in dual-task walking cost, immediately following a single session of tDCS [23]. Although we collected no data concerning the cerebral hemodynamic changes that accompanied changes in performance, the reduction of DTC in the tDCS + ICCT group may provide indirect evidence for improved neural efficiency in the prefrontal cortex.

Our tDCS + ICCT treatment resulted in increased gait speed and reduced gait variability and DTC during dual-task walking. These positive outcomes may have significant clinical implications for older adults with MCI [52]. The improvement in gait speed during dual-task walking we recorded (23.1 cm/s) is clinically meaningful because it exceeds the minimum detectable change value (16 cm/s) [53]. Further, studies have shown that gait variability can predict fall risk and is related to executive function in elderly patients with MCI [54, 55]. It has also been shown that a DTC > 20% can destabilize gait and increase fall risk [56]. In the present study, we recorded a reduction of DTC to 5% following the tDCS + ICCT intervention, suggesting that our program may improve dual-task walking performance and thus reduce fall risk in older adults with MCI.

However, the present study has several limitations. First, we did not have a tDCS-only group, which could have clarified whether the effects were due to tDCS alone or the synergistic effects of the tDCS–ICCT combination. Adding a tDCS-only group to the experimental design in future studies may help address this question. Second, we conducted only pre- and post-intervention assessments; a later follow-up session may be warranted to assess how long the effects of training last after the training period. Third, the small sample size in this study may limit the generalizability of our findings and could affect the statistical power of our results. Fourth. although participants were instructed to prioritize the subtraction task during dual-task performance, we did not record the results of the subtraction task, limiting our ability to explore potential variations in prioritization’s impact on gait performance. Finally, because of inter-individual variability in cortical architecture and the large size of the conventional electrodes we used, the spatial focality of the induced electrical field may not have been optimal. Hence, individual computational modeling may be required in future studies to optimize tDCS dosage and focality.

## Conclusion

Anodal tDCS combined with ICCT had no additional effects compared to ICCT alone in terms of enhancing cognitive function in older adults with MCI, although there were significant cognitive improvements in both groups. However, the tDCS–ICCT combination had a superior effect on dual-task gait performance, suggesting that implementing tDCS as an adjunct to ICCT may provide additional beneficial effects in older adults with MCI. Further studies should improve this protocol and explore the potential of developing it into a nonpharmacological treatment option for alleviating the symptoms of patients with MCI.

## Data Availability

The raw data supporting the conclusions of this article will be made available by the authors, without undue reservation.

## References

[1] Petersen RC (2014). Mild cognitive impairment: a concept in evolution. J Intern Med.

[2] Bahureksa L (2017). The impact of mild cognitive impairment on Gait and Balance: a systematic review and Meta-analysis of studies using Instrumented Assessment. Gerontology.

[3] Pieruccini-Faria F (2020). Mapping associations between Gait decline and fall risk in mild cognitive impairment. J Am Geriatr Soc.

[4] Petersen RC (2018). Practice guideline update summary: mild cognitive impairment: report of the Guideline Development, Dissemination, and implementation Subcommittee of the American Academy of Neurology. Neurology.

[5] Doi T (2014). Cognitive function and gait speed under normal and dual-task walking among older adults with mild cognitive impairment. BMC Neurol.

[6] Olchik MR (2013). Memory training (MT) in mild cognitive impairment (MCI) generates change in cognitive performance. Arch Gerontol Geriatr.

[7] Sherman DS, Durbin KA, Ross DM (2020). Meta-analysis of Memory-Focused Training and multidomain interventions in mild cognitive impairment. J Alzheimers Dis.

[8] Sherman DS (2017). The efficacy of cognitive intervention in mild cognitive impairment (MCI): a Meta-analysis of outcomes on neuropsychological measures. Neuropsychol Rev.

[9] Vermeij A (2017). Prefrontal activation may predict working-memory training gain in normal aging and mild cognitive impairment. Brain Imaging Behav.

[10] Liao YY (2020). Using virtual reality-based training to improve cognitive function, instrumental activities of daily living and neural efficiency in older adults with mild cognitive impairment. Eur J Phys Rehabil Med.

[11] Ge S (2018). Technology-based cognitive training and rehabilitation interventions for individuals with mild cognitive impairment: a systematic review. BMC Geriatr.

[12] Ali N (2022). The effects of Dual-Task training on cognitive and physical functions in older adults with cognitive impairment; a systematic review and Meta-analysis. J Prev Alzheimers Dis.

[13] Liu CL (2022). Effects of Exergaming-Based Tai Chi on cognitive function and dual-Task Gait performance in older adults with mild cognitive impairment: a Randomized Control Trial. Front Aging Neurosci.

[14] Lau LK (2021). Physiological and cognitive determinants of Dual-Task costs for Gait parameters: the Yishun Study. Gerontology.

[15] Lefaucheur JP (2017). Evidence-based guidelines on the therapeutic use of transcranial direct current stimulation (tDCS). Clin Neurophysiol.

[16] Prehn K, Flöel A (2015). Potentials and limits to enhance cognitive functions in healthy and pathological aging by tDCS. Front Cell Neurosci.

[17] Meinzer M (2015). Transcranial direct current stimulation in mild cognitive impairment: behavioral effects and neural mechanisms. Alzheimers Dement.

[18] Gomes MA (2019). Transcranial direct current stimulation (tDCS) in elderly with mild cognitive impairment: a pilot study. Dement Neuropsychol.

[19] Hausman HK (2023). Primary outcome from the augmenting cognitive training in older adults study (ACT): a tDCS and cognitive training randomized clinical trial. Brain Stimul.

[20] Cruz Gonzalez P (2018). Can Transcranial Direct-current stimulation alone or combined with cognitive training be used as a clinical intervention to improve cognitive functioning in persons with mild cognitive impairment and dementia? A systematic review and Meta-analysis. Front Hum Neurosci.

[21] Zhou J (2021). Targeted tDCS mitigates Dual-Task costs to Gait and Balance in older adults. Ann Neurol.

[22] Manor B (2018). Transcranial Direct Current Stimulation May improve cognitive-motor function in functionally limited older adults. Neurorehabil Neural Repair.

[23] Jor’dan AJ (2022). Transcranial Direct Current Stimulation May reduce Prefrontal Recruitment during Dual Task walking in functionally limited older adults - a pilot study. Front Aging Neurosci.

[24] Gonzalez PC, Fong KNK, Brown T (2021). Transcranial direct current stimulation as an adjunct to cognitive training for older adults with mild cognitive impairment: a randomized controlled trial. Ann Phys Rehabil Med.

[25] Lu H (2019). Randomized controlled trial of TDCS on cognition in 201 seniors with mild neurocognitive disorder. Ann Clin Transl Neurol.

[26] Liu CS (2021). Exercise priming with transcranial direct current stimulation: a study protocol for a randomized, parallel-design, sham-controlled trial in mild cognitive impairment and Alzheimer’s disease. BMC Geriatr.

[27] Brunoni AR (2012). Clinical research with transcranial direct current stimulation (tDCS): challenges and future directions. Brain Stimul.

[28] Tsai CF (2012). Psychometrics of the Montreal Cognitive Assessment (MoCA) and its subscales: validation of the Taiwanese version of the MoCA and an item response theory analysis. Int Psychogeriatr.

[29] Miller KM (2009). Is the n-back task a valid neuropsychological measure for assessing working memory?. Arch Clin Neuropsychol.

[30] Ades J, Mishra J. *Systematic review of the longitudinal sensitivity of Precision tasks in Visual Working Memory*. Vis (Basel), 2022. 6(1). 10.3390/vision6010007 PMC8883912 35225968

[31] Chang CC (2010). Validating the Chinese version of the Verbal Learning Test for screening Alzheimer’s disease. J Int Neuropsychol Soc.

[32] Wei M (2018). Diagnostic accuracy of the Chinese Version of the trail-making test for Screening Cognitive Impairment. J Am Geriatr Soc.

[33] Köstering L (2015). Assessment of planning performance in clinical samples: reliability and validity of the Tower of London task (TOL-F). Neuropsychologia.

[34] Dadashi F (2013). Gait and foot clearance parameters obtained using shoe-worn inertial sensors in a large-population sample of older adults. Sens (Basel).

[35] Tiernan C, Schwarz D, Goldberg A (2022). Dual-task cost of the enhanced gait variability index in community-dwelling older adults. Gait Posture.

[36] Fileccia E (2019). Effects on cognition of 20-day anodal transcranial direct current stimulation over the left dorsolateral prefrontal cortex in patients affected by mild cognitive impairment: a case-control study. Neurol Sci.

[37] Hill NT (2017). Computerized cognitive training in older adults with mild cognitive impairment or dementia: a systematic review and Meta-analysis. Am J Psychiatry.

[38] Martin DM (2019). A pilot double-blind randomized controlled trial of cognitive training combined with Transcranial Direct current stimulation for amnestic mild cognitive impairment. J Alzheimers Dis.

[39] Podda MV (2016). Anodal transcranial direct current stimulation boosts synaptic plasticity and memory in mice via epigenetic regulation of Bdnf expression. Sci Rep.

[40] Martin DM (2014). Use of transcranial direct current stimulation (tDCS) to enhance cognitive training: effect of timing of stimulation. Exp Brain Res.

[41] Boripuntakul S (2014). Spatial variability during gait initiation while dual tasking is increased in individuals with mild cognitive impairment. J Nutr Health Aging.

[42] Muir SW (2012). Gait assessment in mild cognitive impairment and Alzheimer’s disease: the effect of dual-task challenges across the cognitive spectrum. Gait Posture.

[43] Kuo HT (2022). Effects of different dual task training on dual task walking and responding brain activation in older adults with mild cognitive impairment. Sci Rep.

[44] Falbo S (2016). Effects of Physical-Cognitive Dual Task Training on executive function and gait performance in older adults: a Randomized Controlled Trial. Biomed Res Int.

[45] Schneider N (2021). Combining transcranial direct current stimulation with a motor-cognitive task: the impact on dual-task walking costs in older adults. J Neuroeng Rehabil.

[46] Manor B (2016). Reduction of dual-task costs by Noninvasive Modulation of Prefrontal Activity in Healthy elders. J Cogn Neurosci.

[47] Wrightson JG (2015). The effect of transcranial direct current stimulation on task processing and prioritisation during dual-task gait. Exp Brain Res.

[48] Holtzer R, Izzetoglu M. *Mild cognitive impairments attenuate Prefrontal Cortex activations during walking in older adults*. Brain Sci, 2020. 10(7). 10.3390/brainsci10070415 PMC7407944 32630216

[49] Doi T (2013). Brain activation during dual-task walking and executive function among older adults with mild cognitive impairment: a fNIRS study. Aging Clin Exp Res.

[50] Nóbrega-Sousa P (2020). Prefrontal Cortex Activity during walking: effects of Aging and associations with Gait and executive function. Neurorehabil Neural Repair.

[51] Mirelman A (2017). Effects of aging on prefrontal brain activation during challenging walking conditions. Brain Cogn.

[52] Montero-Odasso M, Muir SW, Speechley M (2012). Dual-task complexity affects gait in people with mild cognitive impairment: the interplay between gait variability, dual tasking, and risk of falls. Arch Phys Med Rehabil.

[53] Soulard J (2021). Spatio-temporal gait parameters obtained from foot-worn inertial sensors are reliable in healthy adults in single- and dual-task conditions. Sci Rep.

[54] König N (2014). Identification of functional parameters for the classification of older female fallers and prediction of ‘first-time’ fallers. J R Soc Interface.

[55] Mortaza N, Abu Osman NA, Mehdikhani N (2014). Are the spatio-temporal parameters of gait capable of distinguishing a faller from a non-faller elderly?. Eur J Phys Rehabil Med.

[56] Hollman JH (2007). Age-related differences in spatiotemporal markers of gait stability during dual task walking. Gait Posture.

